# Time to Exhaustion at the Respiratory Compensation Point in Recreational Cyclists

**DOI:** 10.3390/ijerph17176352

**Published:** 2020-08-31

**Authors:** Susana Moral-González, Javier González-Sánchez, Pedro L. Valenzuela, Sonia García-Merino, Carlos Barbado, Alejandro Lucia, Carl Foster, David Barranco-Gil

**Affiliations:** 1 Faculty of Sport Sciences, Universidad Europea de Madrid, 28670 Madrid, Spain; susana.moral@universidadeuropea.es (S.M.-G.); javier_entrenamiento_funcional@hotmail.com (J.G.-S.); sonia.garcia@universidadeuropea.es (S.G.-M.); carlos.barbado@universidadeuropea.es (C.B.); alejandro.lucia@universidadeuropea.es (A.L.); david.barranco@universidadeuropea.es (D.B.-G.); 2 Department of Systems Biology, University of Alcalá, 28805 Madrid, Spain; 3 Department of Sport and Health, Spanish Agency for Health Protection in Sport (AEPSAD), 28040 Madrid, Spain; 4 Instituto de Investigación Hospital 12 de Octubre (imas12), 28041 Madrid, Spain; 5 Department of Exercise and Sport Science, University of Wisconsin-La Crosse, La Crosse, WI 54601, USA; cfoster@uwlax.edu

**Keywords:** anaerobic threshold, t_lim_, functional threshold power, endurance performance, cycling, testing

## Abstract

The time to exhaustion (t_lim_) at the respiratory compensation point (RCP) and whether a physiological steady state is observed at this workload remains unknown. Thus, this study analyzed t_lim_ at the power output eliciting the RCP (t_lim_ at RCP), the oxygen uptake (VO_2_) response to this effort, and the influence of endurance fitness. Sixty male recreational cyclists (peak oxygen uptake [VO_2peak_] 40–60 mL∙kg∙min^−1^) performed an incremental test to determine the RCP, VO_2peak_, and maximal aerobic power (MAP). They also performed constant-load tests to determine the t_lim_ at RCP and t_lim_ at MAP. Participants were divided based on their VO_2peak_ into a low-performance group (LP, *n* = 30) and a high-performance group (HP, *n* = 30). The t_lim_ at RCP averaged 20 min 32 s ± 5 min 42 s, with a high between-subject variability (coefficient of variation 28%) but with no differences between groups (*p* = 0.788, effect size = 0.06). No consistent relationships were found between the t_lim_ at RCP and the different fitness markers analyzed (RCP, power output (PO) at RCP, VO_2peak_, MAP, or t_lim_ at MAP; all *p* > 0.05). VO_2_ remained steady overall during the t_lim_ test, although a VO_2_ slow component (i.e., an increase in VO_2_ >200 mL·min^−1^ from the third min to the end of the tests) was present in 33% and 40% of the participants in HP and LP, respectively. In summary, the PO at RCP could be maintained for about 20 min. However, there was a high between-subject variability in both the t_lim_ and in the VO_2_ response to this effort that seemed to be independent of fitness level, which raises concerns on the suitability of this test for fitness assessment.

## 1. Introduction

Various intensity markers or ‘thresholds’ can be used to describe the transition from a steady to non-steady state of oxidative metabolism. These can be assessed during incremental exercise, such as the lactate or ventilatory thresholds (e.g., respiratory compensation point [RCP], also known as second ventilatory threshold) [1] or constant-load tests––notably, to determine the maximal lactate steady state (MLSS) [1] or the critical power (CP) [2].

The RCP, defined as the highest workload that can be sustained before marked hyperventilation occurs due to metabolic acidosis [3,4], has proven to be a performance marker in competitive cyclists [5,6]. Although controversy exists [3], some authors propose that the RCP corresponds to the same workload as other markers of the steady-to-non-steady state transition, such as CP or MLSS [4]. Thus, owing to its usefulness for both testing and training purposes, RCP assessment is a common practice in the sport setting.

Recent research has shown that power output (PO) increases of only 10 W above the MLSS suffice to elicit blood lactate accumulation and a shift in pulmonary ventilation [7]. Similarly, although a steady physiological state has been observed while exercising at the CP, a PO rise of 10% above the CP leads to muscle metabolite accumulation with limited exercise tolerance [8,9]. Estimates of both MLSS and CP seem, therefore, to represent a boundary between intensity domains. However, there is debate on the interchangeability of these landmarks [3,4] and different values of times to exhaustion (t_lim_) have been reported for both estimates—that is, ~60 min for MLSS [10] vs. considerably less (~20–30 min) for CP [11].

The RCP has also been suggested as a landmark of the boundary between heavy and very heavy exercise domains [4]. Different studies have assessed the t_lim_ at the PO corresponding to a key endurance fitness indicator, the peak oxygen uptake (VO_2peak_), yielding t_lim_ values of ~3–4 min [12,13]. However, scarce evidence is available regarding the t_lim_ at the PO corresponding to the RCP (herein simply ‘t_lim_ at RCP’) and whether a steady physiological state is observed at this workload. The primary aim of the present study was to determine the t_lim_ at RCP and the associated VO_2_ response. We also assessed the influence of endurance fitness on the t_lim_ at RCP.

## 2. Materials and Methods

### 2.1. Participants

A convenience sample of sixty healthy male recreational cyclists participated in this study (Table 1). This sample size was considered sufficient as other studies assessing the t_lim_ have included a lower number of participants (i.e., *n* = 14 [12] and *n* = 41 [13]). Participants were free of any disease (e.g., diabetes, asthma) and were not taking any medication. Their training volume was ≥3 h per week (with a cycling experience of ≥3 years). They were instructed to maintain their normal dietary pattern and to refrain from doing intense exercise and consuming ergogenic aids/caffeine 48 h prior to each session. All experiments were performed in accordance with the ethical standards of the Helsinki Declaration. The study was accepted by the Ethics Committee of the University of Alcalá (Madrid, Spain). All participants provided written informed consent.

### 2.2. Procedures

Participants visited the laboratory on two/three occasions separated by 48 h. During the first visit, they performed an incremental maximal cycling test to determine the RCP, VO_2peak_, and maximal aerobic power (MAP). During the second and third visits they performed constant-load tests for the assessment of the t_lim_ at RCP (*n* = 60) and at MAP (*n* = 30), respectively. All the tests were performed using the same cycle ergometer (Ergoselect 200K, Bitz, Germany) and metabolic cart (Ultima Series Medgraphics, Cardiorespiratory Diagnostics, Saint Paul, MN, USA).

The incremental exercise test was performed as explained elsewhere [6]. Briefly, the PO was increased by 25 W∙min^−1^ until exhaustion. The PO corresponding to the RCP was determined as the PO at which an increase in the ventilatory equivalents for both oxygen and carbon dioxide occurred together with a decrease in the end-tidal partial pressure of carbon dioxide [6]. The VO_2peak_ was defined as the highest one-minute average value of VO_2_ reached during the tests. The PO value associated with the VO_2peak_ was defined as MAP.

On the second and third sessions, in a randomized order, participants performed a warm-up (10 min at 75 W with three 30-s sprints at 70% of the PO corresponding to the RCP) followed by a constant-load test to determine the t_lim_ at RCP and MAP, respectively. This warm-up protocol consisting of 5–10 min at submaximal intensities followed by short bursts at higher intensities has been proposed as an effective protocol for maximizing performance [14]. Subjects were required to maintain pedal cadence between 70–90 rpm and the tests were terminated upon volitional exhaustion or when they were not able to maintain 70 rpm. Participants were blinded to the elapsed time and received standardized verbal encouragement.

Gas exchange data were registered during the t_lim_ at RCP test, and the VO_2_ slow component was defined as the occurrence of an increase in VO_2_ > 200 mL·min^−1^ from the third minute of the test to the end of it [15].

### 2.3. Statistical Analysis

Participants were divided into two groups according to their VO_2peak_. Thus, the median VO_2peak_ was calculated for the whole cohort and those with a VO_2peak_ value below or above the median were assigned to a ‘low’- (*n* = 30) or a ‘high-performance’ group (*n* = 30), respectively.

Data are shown as mean ± SD. Differences between the two performance groups were determined using unpaired Student’s *t*-tests. Differences in VO_2_ from the third minute of the t_lim_ test at RCP to the end of it (i.e., for the assessment of the VO_2_ slow component) were determined using paired Student’s t-tests. The magnitude of the differences (effect size, ES) were analyzed through the standardized mean difference (Hedges’ g). ES greater than 0.2, 0.6, 1.2, 2.0, and 4.0 were considered small, moderate, large, very large, and extremely large, respectively [16]. Pearson’s correlation analysis was used to examine the relationship between t_lim_ at RCP and the different endurance fitness markers (RCP, PO corresponding to the RCP, MAP, and VO_2peak_). Correlation coefficients of 0.1, 0.3, 0.5, 0.7, and 0.9 were considered to indicate a small, moderate, strong, very strong, and extremely strong correlation, respectively [16]. The statistical analyses were conducted with a specific software (SPSS 23.0, IBM, Armonk, NY, USA) setting the significance level at 0.05.

## 3. Results

Participants’ demographic data are shown in Table 1. Participants in the low-performance group were older (*p* = 0.015), heavier (*p* = 0.001), and had a higher body mass index (*p* = 0.001) than their high-performance peers. Although the mean values of several fitness markers (VO_2peak_, MAP, and PO corresponding to the RCP) were significantly higher in the high-performance group (all *p* < 0.001, ES > 1.04), the RCP corresponded to a similar relative intensity (% of VO_2peak_ and % of peak power output) in both groups (*p* > 0.05). The average t_lim_ at MAP was 4 min 13 s ± 0 min 58 s, with no differences between the groups (HP: 4 min 4 s ± 0 min 40 s; LP: 4 min 25 s ± 1 min 15 s; *p* = 0.330) and a high between-subject heterogeneity (coefficient of variation [CV] = 23%).

The t_lim_ at RCP averaged 20 min 32 s ± 5 min 42 s, with considerable heterogeneity between subjects (range 11 min 6 s–35 min 15 s, CV = 28%) and non-significant differences between the two groups (20 min 44 s ± 5 min 52 s for LP, and 20 min 20 s ± 5 min 37 s for HP; *p* = 0.788, ES = 0.06). On the one hand, no significant relationship was found between the t_lim_ at RCP and any of the analyzed fitness markers, except for a small correlation with the PO corresponding to the RCP (*r* = −0.291, *p* = 0.024) (Table 2).

**Figure 1:**
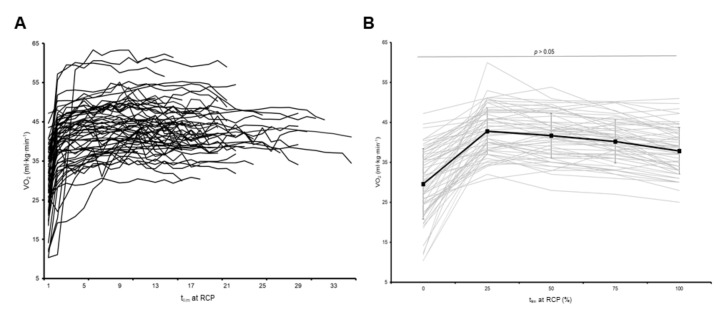
Individual (panel (**A**)) and mean (panel (**B**)) oxygen uptake (VO_2_) response during the time to exhaustion (t_lim_) test at a constant power output (PO) corresponding to the respiratory compensation point (RCP). In panel (**B**), no differences (*p* > 0.05) were observed between the third minute and the end of the test.

The individual and mean VO_2_ responses during the t_lim_ test at RCP are displayed in Figure 1. A possible but non-significant small increase in VO_2_ was observed overall from the third minute of this test to the end of it (from 41.1 ± 7.2 to 42.5 ± 6.6 mL∙kg∙min^−1^, *p* = 0.070, ES = 0.20), although a remarkable heterogeneity was observed, with 20 of 60 participants (33% of total) showing a VO_2_ slow component during the test. A sub-group analysis revealed that the VO_2_ remained steady overall during the test in the high-performance group (from 45.5 ± 5.8 to 46.2 ± 6.1 mL∙kg∙min^−1^, *p* = 0.559, ES = 0.11, unclear differences, with 8 participants [26.6%] showing a slow component). In turn, a small but significant increase in VO_2_ was observed in the low-performance cyclists (from 36.7 ± 5.5 to 38.9 ± 4.7 mL∙kg∙min^−1^, *p* = 0.034, ES = 0.43, possible differences, with 12 participants [40%] showing a slow component).

## 4. Discussion

Previous research has analyzed the t_lim_ and the physiological response at different estimates of the transition from a steady to non-steady state of oxidative metabolism, such as the MLSS or CP. However, the t_lim_ at RCP remained largely unknown to date. The main findings of our study were that the t_lim_ at RCP averages around 20 min regardless of endurance fitness—at least in recreational cyclists—and that this load elicited an overall steady VO_2_ response in most subjects. That being said, there was a high individual variability in the length of the t_lim_ as well as in the occurrence—or not—of the VO_2_ slow component.

To the best of our knowledge, only two studies have previously estimated the t_lim_ at RCP. Bergstrom et al. inferred a t_lim_ value for PO@RCP of ~11 min based on power curve analyses in eight moderately-trained participants [17]. More recently, Pallarés et al. also reported a t_lim_ of ~11 min at the RCP determined using a similar incremental test to that used here (25 W∙min^−1^) [12]. Thus, the t_lim_ at RCP observed in the present study for recreational cyclists of different fitness levels seems to be greater than those reported in previous studies for trained individuals. However, further research is needed to confirm these results as well as to determine the influence of fitness level and of the method used for the assessment of PO at the RCP.

According to our results, the observed average value of the t_lim_ seems to be shorter than that which was previously reported for the MLSS (~60 min) [10,12] but similar to that reported for the CP (~20–30 min) [11,18]. Previous evidence has reported similar PO values for RCP and CP, which in both cases were higher than the MLSS [19]. Some authors support that all of these estimates represent the same physiological phenomenon [4] while others have indicated that both MLSS and CP correspond to a lower intensity than RCP [3,12]. For instance, Pallarés et al. recently reported that the RCP occurred at a higher PO than both MLSS (+22% on average) and CP (+25% on average), whereas MLSS and CP occurred at a relatively similar intensity [12]. Moreover, the t_lim_ at PO@RCP (~11 min) was also markedly lower than that at MLSS (~76 min) [12]. It has been proposed that although MLSS and RCP might correspond to the same VO_2_ value for a given individual, the associated PO might differ due to technical issues (e.g., VO_2_ response time and slow component) with translation of the VO_2_-PO relationship between incremental and constant exercise [4]. Specifically, it has been suggested that determining RCP based on the VO_2_–PO relationship during incremental exercise results in an overestimation of the PO@RCP, which might explain differences between RCP and constant-based estimates (i.e., CP and MLSS) as well as in a non–steady physiological response when exercising at the RCP [4]. Indeed, this overestimation might be particularly relevant in tests with a high ramp rate such as that applied in the present study (i.e., 25 W·min^−1^), which would hinder reaching a physiological steady state in such a short time [20]. On the other hand, the potential overestimation of the MLSS with CP has been explained by the technical error (~5%) inherent to its computation [4]. Thus, further evidence is needed to confirm the equality between RCP, CP and MLSS, as well as to determine the most accurate protocol for the assessment of these physiological markers.

Also noteworthy is our finding of a heterogeneous VO_2_ response during the t_lim_ test at RCP, with one third of the participants showing a meaningful increase in VO_2_ (i.e., >200 mL·min^−1^) and thus the slow component phenomenon, while the remainder of the subjects showed a more steady response. Indeed, although statistical differences were found between the two study groups regarding the VO_2_ response to the t_lim_ test (i.e., steady in the high-performance group but non-steady in the low-performance group) the number of participants in each group presenting with a meaningful increase in VO_2_ during the test was similar. Similarly, it has been reported that exercising at—but not 5–10% above—the CP results in a steady physiological state [8,9], although other authors failed to find a steady-state response when exercising at the CP [11,18]. In any case, it must be noted that, in addition to the individual variability we found, a steady VO_2_ response at PO at the RCP might not necessarily reflect an overall steady physiological state, as supported by a recent study reporting steady VO_2_ kinetics when exercising above the MLSS despite the presence of metabolic perturbations (e.g., blood lactate accumulation) [7].

The present findings can also be discussed in relation with another estimate of the transition between the steady and non-steady domains which has grown in popularity in recent years—the functional threshold power (FTP). FTP corresponds to the highest mean PO that athletes can sustain for around one hour, which, for the sake of practicality, is usually determined by subtracting 5% from the mean PO achieved during a 20-min test at the maximum tolerable intensity [21]. In accordance with the t_lim_ observed in the present study, a strong agreement between RCP and the mean PO during a 20-min test has been previously reported [22]. Our group recently demonstrated a good agreement between the FTP and the lactate threshold determined with the ‘D-max’ method [23]. The D-max has also been reported to have a strong correlation (*r* = 0.97) with the MLSS [24]. Recent research has shown that the FTP presents a strong agreement with the MLSS [25]. Moreover, it has been reported that the FTP-associated PO can be maintained for ~51 min before exhaustion [26], which is similar to the t_lim_ reported for the MLSS (~50–60 min) [10]. These results would, overall, support that the mean PO during a 20-min test might be reflective of the RCP, and that 95% of that PO might be similar to the MLSS. However, a recent study reported that although the FTP was strongly correlated to the MLSS (*r* = 0.95), the former corresponded to a significantly higher PO [27]. Notably, we recently found that the mean PO obtained during a 20-min test strongly correlated with the RCP in highly-trained cyclists, but significantly higher values were found for the PO at the RCP compared to the PO that was sustainable during the 20 min (bias ~12%) [28]. Lillo-Beviá et al. also reported higher PO values at the RCP than those obtained during a 20-min test [27]. These findings should be confirmed in future studies.

Our group also recently observed that the level of agreement between the mean PO during a 20-min test at the maximum possible intensity and the lactate threshold was dependent on participants’ fitness status [23], which is why we hypothesized for the present study that the t_lim_ at RCP would increase with endurance fitness level. The present results seem, however, to refute this hypothesis, and suggest that a 20-min test could be used to obtain an approximation of the RCP in recreational cyclists of different levels (VO_2peak_ 40–60 mL∙kg∙min^−1^). Notwithstanding, the wide between-subject heterogeneity (23%) observed for the t_lim_ at RCP*—*which is similar to that previously reported for the t_lim_ at the VO_2peak_ (i.e., 25%) [29]*—*must be noted. Moreover, the lack of differences between the two performance groups in the %VO_2peak_ at which the RCP occurred might have contributed to the lack of differences in the t_lim_, although no significant correlation between markers was found in the present study. We cannot rule out that different results might have been found in highly trained cyclists.

Finally, as the physiological response when exercising at a fixed relative intensity (e.g., at a given %VO_2peak_) is highly heterogeneous between individuals [30], it has been proposed that prescribing exercise intensity relative to individually-determined physiological threshold measurements might homogenize the elicited stress and reduce the individual variation in metabolic responses. In this regard, the lack of influence of endurance fitness in the t_lim_ supports this concept, but in turn the high between-subject heterogeneity observed in both performance (CV = 23% for t_lim_) and physiological responses (with 33% of the subjects presenting a non-steady VO_2_ response) raises concerns on the suitability of this strategy.

## 5. Conclusions

A t_lim_ test at RCP-associated PO seems to elicit an overall steady VO_2_ response and can be maintained for about 20 min before exhaustion in recreational cyclists. However, there is a high individual variability in both the t_lim_ at RCP (CV = 23%) and the VO_2_ response during the t_lim_ test itself (with 33% of the subjects presenting a non-steady VO_2_ response), that seems to be independent of athletes’ endurance fitness level, which raises concerns on the suitability of this test for performance assessment or for the guidance of training intensity prescription.

## Figures and Tables

**Table 1:** Descriptive data of all subjects, and comparative analysis between low- and high-performance cyclists.

Variable	All Subjects (*n* = 60)	Low-Performance Group (*n* = 30)	High-Performance Group (*n* = 30)	*p*-Value	ES
Age (years)	37 ± 9	40 ± 7	34 ± 11	0.015	0.65
Weight (kg)	78 ± 8	81 ± 7	74 ± 8	0.001	0.93
Height (cm)	177 ± 7	178 ± 6	177 ± 8	0.822	0.14
BMI (kg∙m^−2^)	24.6 ± 2.3	25.6 ± 1.8	23.6 ± 2.4	0.001	0.93
VO_2peak_ (mL∙kg^−1^∙min^−1^)	50 ± 8	44 ± 3	56 ± 6	<0.001	2.52
MAP (W∙kg^−1^)	4.20 ± 0.70	3.73 ± 0.38	4.86 ± 0.56	<0.001	2.36
MAP (W)	323 ± 40	302 ± 35	344 ± 33	<0.001	0.55
RCP (W∙kg^−1^)	3.27 ± 0.57	2.90 ± 0.41	3.63 ± 0.47	<0.001	1.65
RCP (W)	251 ± 37	234 ± 36	268 ± 29	<0.001	1.04
RCP (% VO_2peak_)	85 ± 6	85 ± 6	86 ± 6	0.412	0.16

Data are mean ± SD. Abbreviations: BMI, body mass index; ES, effect size; MAP, maximal aerobic power output; VO_2peak_, peak oxygen uptake; RCP, respiratory compensation point.

**Table 2:** Association between different physiological/performance variables and the time to exhaustion (t_lim_) at the respiratory compensation point (RCP).

Variable	*r*	*p*-Value
t_lim_ at MAP (min)	−0.193	0.306
RCP (mL∙kg∙min^−1^)	−0.044	0.737
RCP (% VO_2peak_)	−0.184	0.160
VO_2peak_ (mL∙kg∙min^−1^)	0.030	0.822
MAP (W∙kg^−1^)	−0.016	0.903
MAP (W)	−0.119	0.367
RCP (W∙kg^−1^)	−0.172	0.190
RCP (W)	−0.291	0.024 *

Abbreviations: MAP, maximal aerobic power output; RCP, respiratory compensation point; VO_2peak_, peak oxygen uptake. * indicates a significant association (*p* < 0.05).
